# Djinn Lite: a tool for customised gene transcript modelling, annotation-data enrichment and exploration

**DOI:** 10.1186/1471-2105-7-33

**Published:** 2006-01-23

**Authors:** Erdahl T Teber, Edward Crawford, Kent B Bolton, Derek Van Dyk, Peter R Schofield, Vimal Kapoor, W Bret Church

**Affiliations:** 1 School of Medical Sciences, University of New South Wales NSW 2052, Australia; 2 EBM Pty Ltd, Level 6, 110 Sussex Street, Sydney, NSW 2000, Australia; 3 NSW Ministry for Science and Medical Research, GPO Box 5341, Sydney NSW 2001, Australia; 4 Neurobiology Division, Garvan Institute of Medical Research, Sydney NSW 2010, Australia; 5 Faculty of Pharmacy, University of Sydney NSW 2006, Australia; 6 Prince of Wales Medical Research Institute, Sydney NSW 2031, Australia; 7 Department of Medicine and Pharmacology, University of Western Australia, Crawley WA 6009, Australia

## Abstract

**Background:**

There is an ever increasing rate of data made available on genetic variation, transcriptomes and proteomes. Similarly, a growing variety of bioinformatic programs are becoming available from many diverse sources, designed to identify a myriad of sequence patterns considered to have potential biological importance within inter-genic regions, genes, transcripts, and proteins. However, biologists require easy to use, uncomplicated tools to integrate this information, visualise and print gene annotations. Integrating this information usually requires considerable informatics skills, and comprehensive knowledge of the data format to make full use of this information. Tools are needed to explore gene model variants by allowing users the ability to create alternative transcript models using novel combinations of exons not necessarily represented in current database deposits of mRNA/cDNA sequences.

**Results:**

Djinn Lite is designed to be an intuitive program for storing and visually exploring of custom annotations relating to a eukaryotic gene sequence and its modelled gene products. In particular, it is helpful in developing hypothesis regarding alternate splicing of transcripts by allowing the construction of model transcripts and inspection of their resulting translations. It facilitates the ability to view a gene and its gene products in one synchronised graphical view, allowing one to drill down into sequence related data. Colour highlighting of selected sequences and added annotations further supports exploration, visualisation of sequence regions and motifs known or predicted to be biologically significant.

**Conclusion:**

Gene annotating remains an ongoing and challengingtask that will continue as gene structures, gene transcription repertoires, disease loci, protein products and their interactions become moreprecisely defined. Djinn Lite offers an accessible interface to help accumulate, enrich, and individualise sequence annotations relating to a gene, its transcripts and translations. The mechanism of transcript definition and creation, and subsequent navigation and exploration of features, are very intuitive and demand only a short learning curve. Ultimately, Djinn Lite can form the basis for providing valuable clues to plan new experiments, providing storage of sequences and annotations for dedication to customised projects. The application is appropriate for Windows 98-ME-2000-XP-2003 operating systems.

## Background

There remains intense interest in the annotation of genomes, and work is continuing to be done to elucidate all of the human genes, its haplotypes [1,2] and its transcriptome [3,4]. It is estimated approximately 50% of the human transcriptome is not yet annotated [3]. According to the CCDS project, as at March, 2005, just over 13,000 genes can be reliably said to code for protein [5], which represents approximately half of the human genes [6]. Inconsistencies in gene annotations arise due to different human genome sequence database centers employing different methods for identifying the locations of genes and generating coding sequences [5], specifically from different computational methods and gene-finding programs. "Ab initio" gene finding programs detect genes by looking for distinct patterns that define where a gene begins and ends. Alternatively, comparative gene finding programs look for genes by comparing segments of sequence with those of known genes and proteins [6]. Current gene prediction algorithms also focus largely onpredicting coding regions and less on untranslated regions [7,8]. Gene finding computational methods alone are simply insufficient to generate accurate gene structures. Providing accurate gene annotations will take the coordinated efforts of experimentalists and computational biologists to learn from the inconsistencies between gene structures generated by manual curation and automated methods [9].

Also, limited numbers of tissue-specific EST and mRNA sequences deposited into public databases, as well as different cDNA construction protocols may miss tissue-specific transcript constructs [10]. Again, biological experiments may be necessary to confirm any transcript constructions, but programs which can suggest best-guesses and ranges of transcript options could be extremely valuable.

The need for manual gene curating is also necessitated due to errors and inaccuracies that may exist in the main sequence databases [11,12]. It is not uncommon for different sources to give different names to the same regions (e.g. Exons), particularly between literature and genome database sources (e.g. PTGS1/COX1 gene) [13-15]. These situations make manual curating all the more important, as biologists attempt to resolve inconsistencies.

In the past 30 years, genetic studies of multifactorial human diseases have identified approximately 50 genes and their allelic variants. However, it is estimated that there are likely to be hundreds of susceptibility loci that increase the risk for each common disease [16]. Therefore, annotating of genes remains ongoing and represents a challenging task that will be driven forward continually as the human gene structures and disease loci become more precisely defined. Current efforts are underway to develop a haplotype map of the human genome [1,2]. The haplotype map or "HapMap" aims to provide researchers with information to find genes and genetic variations that affect health and disease. Manual annotators will play a critical role cataloguing how different components interact and contribute to biological processes, diseases and physiological complexity. Thus, investigators require tools to be able to store, test and analyse the combinations of alleles experimentally observed to be inherited as units from DNA/polymorphism screenings.

It is estimated that 75% of alternative splicing events change the protein coding sequence [17,18]. It is thought that approximately a third to half of all human genes produce multiple transcript variants [19]. Alternative splicing can often produce protein isoforms with different domain compositions and motifs [20]. It will be vital to use tools to be able to model variant transcripts to test for splice-site plasticity and disease forming missplicing events [21].

Along with our understanding of alternate splicing events and post-translational modification it is likely that biologists with expertise in specific genes will continue to curate annotations and will benefit from "clue" providing visualisation tools to perform targeted experiments on regions of eukaryotic genomes. As sequences are accumulating at a great rate, biologists are also required to assimilate sequence related information from many diverse sources (Table 1). Moreover, putative or customised annotations are always required by individual laboratories, the implications of which can be further tested.

### Comparisons

A number of sophisticated and powerful sequence annotation and visualisation tools are available including ARTEMIS [22], SeqVISTA [23], and Genotator [24,25]. These tools principally focus on features that are related to segments of nucleotide sequences or small genomes, translated amino acid sequences and their annotations. Many of the features that are necessary for visualising sequences such as ease of navigation, colour coding, and dynamic linking of macro level depictions with detailed sequences exist in these programs. They also contain additional integrated functionality which can be useful to experienced bioinformaticians, including exon predicting, dbEST searches, protein secondary structure predictions and others [22-24].

Genome browsers on the other hand, are suited to large scale annotation and analysis of genomes and include UCSC's Genome Browser [26], ENSEMBL project viewer [27], NCBI Map Viewer and GeneViTo [28]. UCSC's Genome Browser can display requested portions of genomes by zooming in/out to any scale, together with many aligned annotation tracks, including known genes, predicted genes, ESTs, mRNAs, CpG islands, assembly gaps and coverage, chromosomal bands, cross-species homologies, and tracks that have been deposited by others. Users can add and view their own custom tracks, however, this can require users to place annotations in formatted files before uploading into the browser.

NCBI Human Map viewer also has an additional function called Model Maker which is able to show the exons provided by GenBank, mRNAs, ESTs, and gene predictions. However, the numbering and alignments between transcript and genomic sequence, between transcript and protein are non-intuitive, requiring jumping between screens to obtain position number associations. Another key limitation of web-based visualisation and annotation tools is the available printing options. These are limited to printing only what is available on the page or images, and can make copying/pasting sequences and alignments cumbersome.

The number and type of annotations can vary and arranging annotations in a non-confusing manner is paramount for non-bioinformatic conversant biologists. Ultimately, excessive functionality, formatting of input files, genome wide analysis, and inflexible printing can be overwhelming for biologists whose key focus would be to judiciously conduct wet laboratory experiments on their gene of interest. Thus, unneeded complexities related to gene annotations need to be hidden from view, and software tools need to be less complicated in an effort to help in integrating, storing and visualising annotations as biologists gradually learn more about their gene of interest.

We have developed Djinn Lite for those users not requiring significant bioinformatics skills in customising gene/transcript annotations. The application is appropriate for Windows 98-ME-2000-XP-2003 operating systems.

## Implementation

### Input sequence, defining transcripts and coding regions

**Figure 1:**
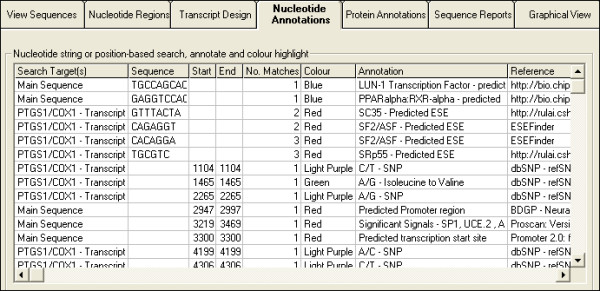
**Tab designed workflow of Djinn Lite**. Prostaglandin-endoperoxide synthase (PTGS1) plays a major role in prostaglandin biosynthesis. Commonlyreferred to as Cyclooxygenase 1 (COX1), PTGS1 is a target for aspirin and other non-steroidal anti-inflammatory drugs (NSAIDs), in particular, for reducing platelet aggregation (GenBank: NT_008470 Chromosome 9 genomic contig – nucleotides 32,450,650-32,481,650). This figure displays a section of the nucleotide annotations page showing a customised list of PTGS1 gene annotations, including predicted promoter elements, transcription factor binding regions, enhancer splicing elements and known SNPs.

Djinn (pronounced jeen or jēn) Lite has been designed in keeping with a novice gene annotator's general workflow. This is facilitated by the use of "tabs" (analogous to dividers in a notebook or the labels in a file cabinet). A single application window contains six (6) tabs each corresponding to a single page, these are: "View Sequences", "Nucleotide Regions", "Transcript Design", "Nucleotide Annotations", "Protein Annotations", "Sequence Reports", and "Graphical View" (Figure 1).

Djinn Lite invokes a wizard to allow for the input of a raw nucleotide sequence. The raw nucleotide sequence can be genomic, pre-mRNA, mRNA or partial or complete protein coding region (CDS) sequences. For example, raw nucleotide sequences can be obtained from any sequence database including NCBI Entrez Gene [14], Ensembl [15], GeneCards [29] and Celera [30] and then cut-and-paste into Djinn Lite's main sequence input text form as part of the initial wizard.

Djinn Lite uses the term "Nucleotide Regions" to describe core transcriptional regions, such as exons, 5' and 3' untranslated regions. Upon the input of a main sequence key regions may be defined by either providing a start and end nucleotide position or selecting the nucleotides using the mouse by click-and-dragging. Alternatively, regions may be assigned after carrying out a nucleotide string search match. Textual information describing the source of the information may be added to a "Reference" entry field pertaining to a defined "Nucleotide Region".

Transcripts can be generated by selecting from a list of previously defined transcriptional regions referred to as "Nucleotide Regions". When an mRNA transcript is created, a check box can be used to avoid translating of flanking regions such as 5' and 3' UTRs. The checkbox by default remains checked to allow the entire construct to be translated as in the case of a coding sequence (CDS).

### Feature annotation and colour highlighting

Annotating and colour highlighting of particular nucleotide or translated protein sequence can be carried out by either conducting a sequence search or by providing a sequence start and end position. Adding annotations using the "Highlight Nucleotides" enables users to carry out searches against a list of previously defined transcripts, CDSs, including the main input sequence (e.g. genomic sequence). There are 15 colours to choose from for the highlighting of sequence regions and macro level depictions (a bar that displays colour annotations over graphical box representations of the gene, transcripts, and proteins). Thus, colour highlights can be overlaid on top of sequence regions which can include; 5' transcriptional control elements, promoters, translational control elements, start and stop codons, 3' polyadenylation signals, binding sites for transcription factors, splicing enhancer/silencing elements, polymorphic variants, SNPs, mutations, microRNA and small interfering RNA (siRNA) targets, RNA editing sequences, protein domains, motifs and protein binding regions, PCR covered regions, putative regions or regions requiring experiment validation (Figure 1 and Table 1). Users have the discretion and flexibility to make any annotation of their choosing. Also, grey colour highlights are automatically generated by Djinn Lite where there are overlapping colour coded annotations along a sequence. Each annotation also enables the attachment of a textual description which appears in the legends in both the "Sequence Reports" and "Graphical View" pages.

### Graphical representation and viewing sequences

Djinn Lite uses multiple rows or tracks [31] for handling the complexity of genomic sequence annotations enabling numerous annotations and incorporates a multiple dimensional data space (sequence, transcript regions, features). The "View Sequences" tab is the "working display" window showing the sequence(s) (base pairs) in their entirety viewable by horizontal scrolling bar.

The user can toggle between the macro view identified as "Graphical Overview" and the "View Sequence". The "Graphical Overview" provides the user with a high level picture representation of the gene, transcripts and protein.

The "Graphical Overview" was designed to be particularly useful for gaining an overview of the physical size of the gene map and its associated transcripts, in terms of the relative sizes of the introns, exons, and the density of features along these maps. The sizes of the transcripts and their associated proteins are scaled relative to each other. Thus, an inspection of key global differences between transcripts can provide clues to dissimilarities in transcriptional regions and protein domains or motifs.

Within the "Graphical Overview" there are two sections, the genomic view and the transcripts view. Within the genomic view boxes represent exons, narrow lines represent introns or non-genic regions, and below is an annotation ruler designed to display colour bars to assist in featured annotations alongside their corresponding relative locations along the genomic map. Djinn Lite is also able to depict "overlapping exons" and "overlapping untranslated regions", as can occur due to the plasticity of splice-site selection [21], as dark green coloured boxes. The transcripts view depicts all of the defined transcripts, where boxes represent untranslated, exons or coding regions. An annotation ruler displays colour bars to feature annotations alongside their corresponding relative location along the transcript. Also, the translated transcript is displayed as an outlined box and can be overlaid with colour code bars to correspond to annotations relative to the protein.

**Figure 2:**
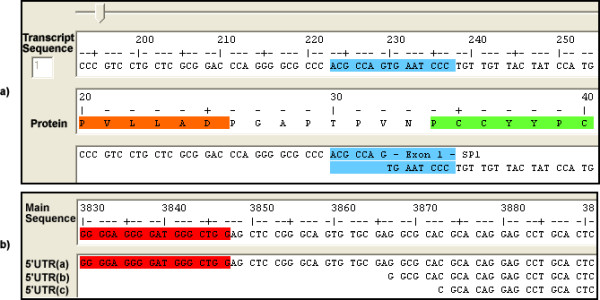
**Viewing sequences and position numbering of associated regional segments**. a) The Exon 1/Exon 2 splice junction of transcript 1 (SP1) alongside the translated sequence. Annotated regions have the colour highlights. Colours red, green, and aqua, respectively, refer to a putative signal peptide, an Epidermal Growth Factor domain, and the splice site junction between Exon 1/Exon2. b) The three varieties of 5'UTRs ([GenBank: NM_080591.1]  and two computationally derived alternate splice variants from AltSplice [32]) aligned to the PTGS1 gene sequence (main sequence). Alternate 5' sequences can occur due to differential regulation of upstream promoters and splicing factors, giving rise to different transcription-start sites and splice donor/or acceptor sites [21, 33]. Mfold program [34] predicts a hair-pin RNA secondary structure within 5' UTR(a), represented in red. Secondary structures in the 5'UTR can modulate translation efficiency.

More critical inspection of features (regions and annotations) is facilitated by allowing for flexible viewing combinations via zooming and horizontal scroll bars which are located at the bottom of every genomic and transcript graphical representation. Clicking on any of the graphically displayed boxes (exon, untranslated regions) or narrow lines (non-genic or intronic regions) will invoke the "View Sequences" tab and call up in real-time the sequence corresponding to the selected region (Figure 2).

The user is able to maintain context between the "Graphical Overview" and "View Sequences", as the "View Sequences" tab, either displays transcript regions in context to its genomic DNA or a protein in context to its transcript. This allows the user to view sequence alignments and numbering of nucleotide and amino acid positions in an integrated context.

### Previewing/printing/exporting sequences and picture representations

**Figure 3:**
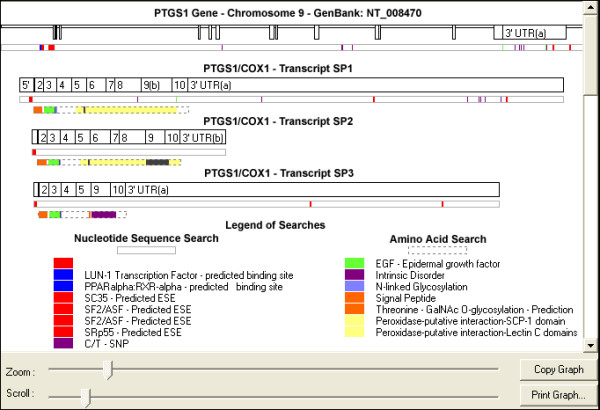
**Graphical Representation**. A representation of the PTGS1 gene (approx. 30 kb) and three (3) modelled gene products, transcript SP1 to SP3. The sizes of the transcripts and their associated proteins are scaled relative to each other, providing visual insights into the differences between transcripts, and clues to dissimilarities in transcriptional regions and protein domains or motifs. Boxes represent exons and untranslated regions and narrow lines represent introns or non-genic regions. An annotation ruler displays colour bars that feature annotations alongside their corresponding relative location along the transcript. Translations of transcripts are displayed as outlined boxes and are overlaid with colour code bars of annotations relative to the protein. Annotation legends are depicted at the bottom.

The user has two main options for the printing of annotations. These are previewing or printing of high level picture representations and the detailed sequences and annotations. The first option, the "Graphical View" tab, has the same functionality as the "Graphical Overview" window, but instead enables printing of high level picture representations of genomic, pre-mRNA, mRNA, partial or complete protein coding region (CDS) sequences, together with the colour annotations and legends (Figure 3). The size and viewing pose of each picture object (gene, transcripts, CDSs) can be varied by clicking on any part of the object and then by zooming/scrolling. The current viewing pose can then be copied/pasted for reporting purposes.

The second option, the "Sequence Reports" tab, enables printing of sequences alongside aligned regions, amino acid sequences, with colour-codes overlaid on the sequences. This results in a table representation of the sequences, including a legend for the annotations at the end of the report. Printing is context specific, i.e. transcript regions are aligned in context to its genomic DNA or optionally a protein is aligned in context to its transcript. The width of the table can be modified, thus allowing users to have a large range of sequence lengths to aid their viewing requirements. It is then possible to copy/paste, which provides for report writing and ultimately for publication purposes.

Djinn Lite also allows for exporting of gene, transcript, CDS, transcriptional nucleotide regions (exons and untranslated regions) and protein sequences in FASTA format. All accumulated sequences, transcript models and annotations can be saved as a text formatted file. This text file can be easily copied into Microsoft Excel for further manipulation or analysis.

### Software design limitations

Once a main sequence entry has been initially processed in Djinn Lite the nucleotide sequence state remains static and subsequent nucleotide changes (addition/deletions/substitutions) within a saved gene configuration are not allowed. Thus, Djinn Lite is not designed for automatic updating of downstream gene product sequences when alterations are made to the inputted nucleotide sequence. Real-time changing of nucleotides at the main sequence level would be a useful feature in helping to observe the effects of nucleotide changes at downstream levels, including changes to splicing, domains/motifs, and amino acid changes. This would force the program to respond to a multitude of subsequent effects, including changes to regions and transcript variants. This feature was not implemented in real time to prevent the user interface from becoming too complex, as it could potentially yield multiple user notifications to highlight many of the subsequent downstream sequence alterations. However, a separate Djinn session and file can be set up to accommodate for different sequence variations of a gene or sequence entry. For example, Djinn Lite can be used for haplotype mapping, where each distinct haplotype (distinct set of polymorphic variations which are inherited as a unit) can be set up as a separate Djinn file.

Again, in an effort to maintain the simplicity of the Djinn Lite's user interface, some features which are biologically relevant to mRNA were not implemented. We believed that these features were not critical to the main emphasis and utility of Djinn Lite, which was the ease of use, uncomplicated transcript modeling, annotating and visualising. For example, thymine (T) is not replaced for uracil (U) when DNA is transformed to RNA. This aspect may be more important for programs that provide RNA secondary structural analysis, as uracil and thymine have different hybridization properties. This was not part of Djinn Lite's design scope. Djinn Lite avoids accommodating for addition of multiple adenosine nucleotides onto the 3' end of defined transcripts (polyadenylation) for subsequently defined and transformed downstream sequences. Likewise, RNA editing such as substitution or deletion or insertion editing are avoided. Also, Djinn Lite does not permit loose sequence alignments.

## Conclusion

Djinn Lite represents a tool for the process of "annotation data enrichment", which involves the incremental gathering, qualifying and experimental verification of both putative and documented gene sequence annotations. Djinn Lite provides the ability to display annotations, easy to follow numbering of aligned sequences, creation of alternative transcript models using novel combinations of exons, as well as offer flexible printing options for annotated sequences and gene/transcript maps. The interface is intuitive, requiring only a short learning curve, helping to quickly accumulate and individualise sequence information on genes and their flow on products, including sequence annotations relating to transcriptional/translational regulation, post translational modifications and protein interactions. Djinn Lite can provide storage of gene annotations for personalised projects on particular genes of interest and therefore be the basis of valuable clues to plan new experiments so that the needs of biologists whose key concern is to judiciously plan and conduct experiments are met. Ultimately, extensive use of such a tool can help to improve the accuracy and comprehensiveness of genome wide annotations. Additionally, Djinn Lite may be a useful teaching aid to support the learning of undergraduate students on topics related to gene structure.

## Availability and requirements

Project name: **Customised gene transcript modeling, annotating and exploring**

Project name home page: ****

Operating system: **Microsoft 98/ME/2000/XP/2003**

Programming language: **Visual Basic Version 6**

Other requirements: **None**

Licenses: **Executable is freeware**

Any restrictions to use by non-academics: **None**

## Authors' contributions

ETT was responsible for the original concept and design, programming, testing and drafting of the manuscript; KBB programmed at the initial stage to create a viable program; EC was involved in design improvements of the user interface and graphics, a substantial overhaul of the data structures, and programming; DVD was involved in the design improvements; PRS provided experimentalist perspective on the biology of alternate splicing; VK provided experimentalist perspective on the biology of alternate splicing and testing; WBC was responsible for drafting of the manuscript, design improvements and substantial testing.

## Figures and Tables

**Table 1:** A sample of sources available on experimentally, computationally derived gene annotations and sequence pattern discovery programs

**Annotation Type**	**Source**	**Reference**
Alternative splicing	ASD AltSplice and AltExtron – Computationally derived splicing events. AEdb – splicing events manually generated from the literature.	[32]
	Swiss-Prot/TrEMBL	[35-37]
Polymorphic sequence variations	dbSNP	[38]
Predicting RNA secondary structures	Mfold	[34]
Predicting protein motif/domains/structural similarities	SMART	[39]
Human gene mutations	OMIM HGMD	[40] [41]
Predicting transcription factor binding sites	MAPPER	[42]
Predicting exonic splicing enhancer sites	ESEFinder RESCUE-ESE	[43] [44]
Predicting promoter elements	SIGNAL SCAN FirstEF	[45]
Post-translational modifications	Swiss-Prot/TrEMBL	[35-37]
Predicting post-translational modifications	NetAcet – N-terminal acetylation in eukaryotic proteins. NetNGlyc – N-linked glycosylation sites in human proteins. NetOGlyc – O-GalNAc (mucin type) glycosylation sites in mammalian proteins. NetPhos – Serine, threonine and tyrosine phosphorylation sites in eukaryotic proteins. ProP – Arginine and lysine propeptide cleavage sites in eukaryotic protein sequences. YinOYang – O-(beta)-GlcNAc glycosylation and Yin-Yang sites (intracellular/nuclear proteins). All above web server programs can be found at	[46] [47] [48] [49]
Protein-protein interaction data	InterDom BIND DIP MINT	[50] [51] [52] [53]

Some examples of web based sources providing free sequence related annotation data and "biologically significant" sequence pattern discovery programs. Most of these sources can provide specific annotations upon inputting of a gene/transcript/protein sequence, or the name of the gene/protein, along with the source organism. It is the thesis of our work that care needs to be taken to assess the quality of all sources of information. This is not intended to be an exhaustive list, but highlights sources used for the examples included in this paper.
